# Supplementary figures and images for: Comparative Physiological and Transcriptomic Analyses of Two Contrasting Pepper Genotypes under Salt Stress Reveal Complex Salt Tolerance Mechanisms in Seedlings
Source: Int J Mol Sci. 2022 Aug 26;23(17):9701. doi: 10.3390/ijms23179701 (PMC9455954; doi:10.3390/ijms23179701)

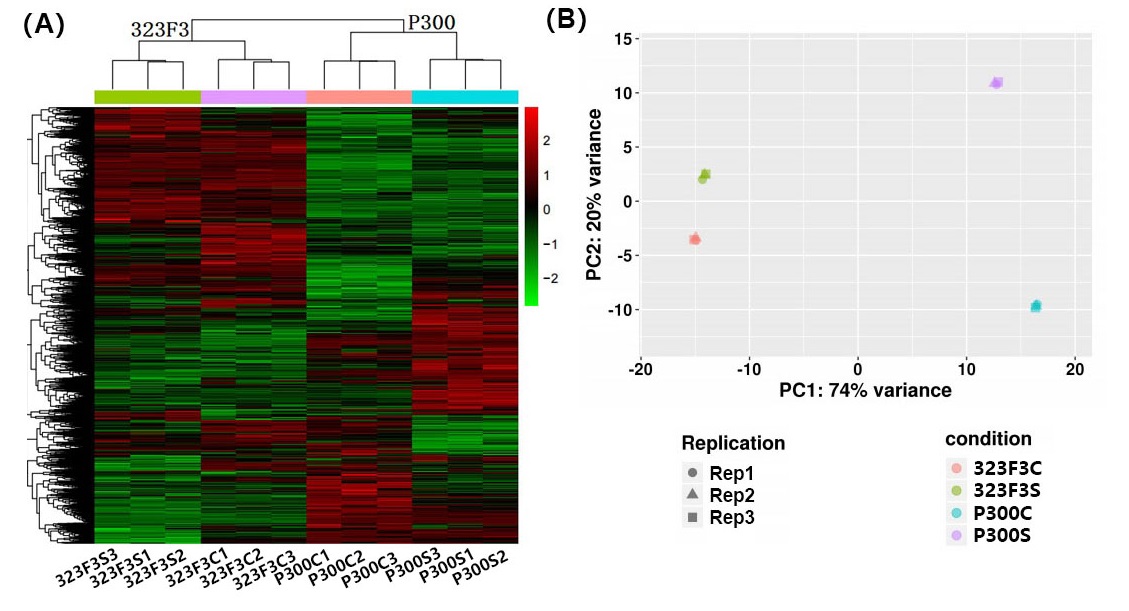

Supplement: Supplementary file 1 [file ijms-23-09701-s001.zip › Figure S1.jpg]

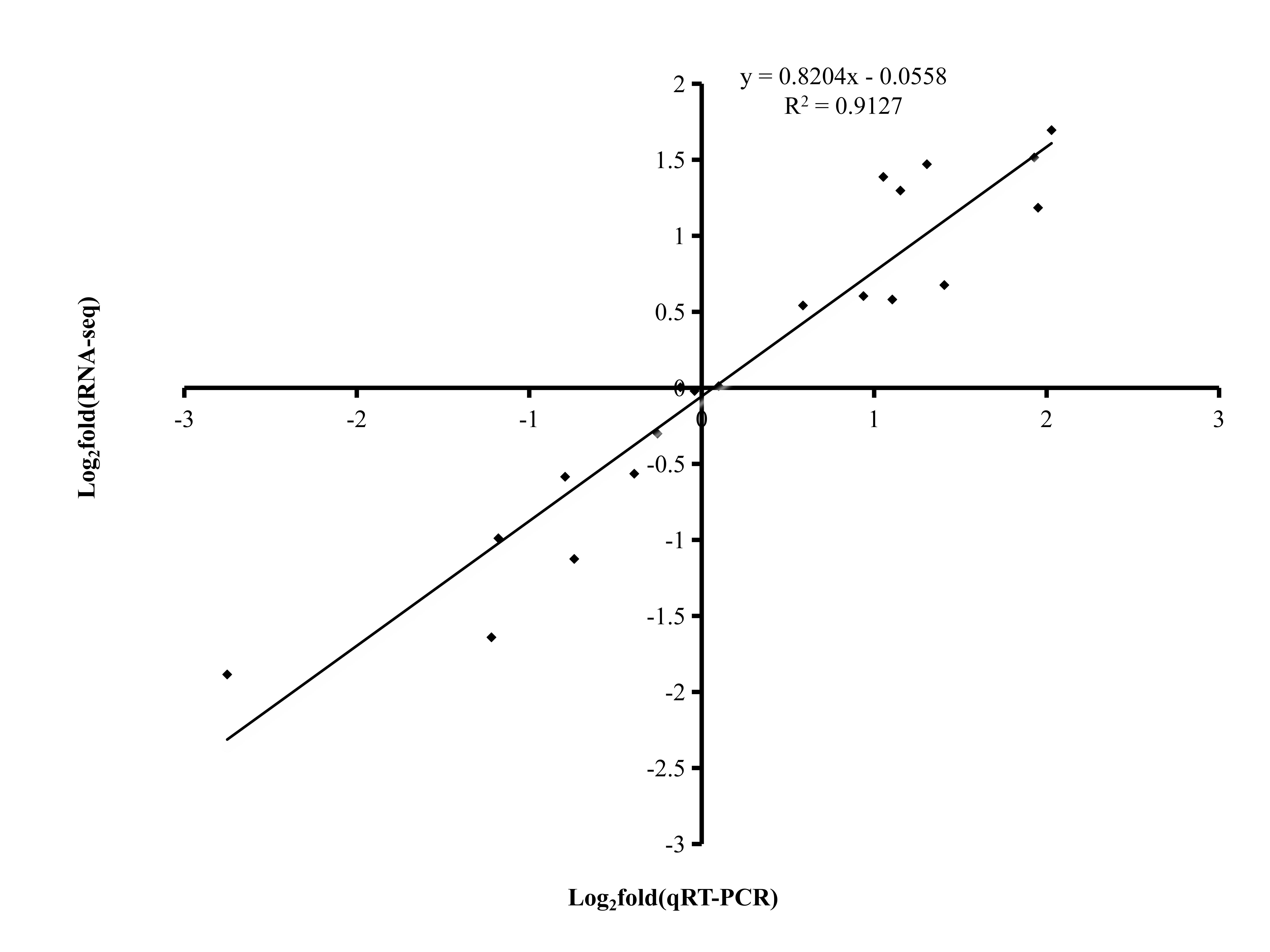

Supplement: Supplementary file 1 [file ijms-23-09701-s001.zip › Figure S2.jpg]
